# DCE-MRI and DWI can differentiate benign from malignant prostate tumors when serum PSA is ≥10 ng/ml

**DOI:** 10.3389/fonc.2022.925186

**Published:** 2022-12-12

**Authors:** Hongmei Sun, Fengli Du, Yan Liu, Qian Li, Xinai Liu, Tongming Wang

**Affiliations:** ^1^ Department of Magenetic Resonance Imaging (MRI), Henan Province Hospital of Traditional Chinese Medicine (The Second Affiliated Hospital of Henan University of Chinese Medicine), Zhengzhou, China; ^2^ Henan University of Chinese Medicine, Zhengzhou, China; ^3^ School of Medical Engineering, Xinxiang Medical University, Xinxiang, China

**Keywords:** prostate cancer, prostate-specific antigen, PI-RADS, dynamic contrast-enhanced magnetic resonance imaging, diffusion-weighted imaging

## Abstract

**Background:**

This study investigated the diagnostic utility of dynamic contrast-enhanced magnetic resonance imaging (DCE-MRI) and diffusion-weighted imaging (DWI) parameters for distinguishing between benign and malignant prostate tumors when serum prostate-specific antigen (PSA) level is ≥10 ng/ml.

**Methods:**

Patients with prostate cancer (PCa) and benign prostatic hyperplasia (BPH) with serum PSA ≥10 ng/ml before treatment were recruited. Transrectal ultrasound-guided biopsy or surgery was performed for tumor classification and patients were stratified accordingly into PCa and BPH groups. Patients underwent DCE-MRI and DWI scanning and the transfer constant (K_trans_), rate constant (K_ep_), fractional volume of the extravascular extracellular space, plasma volume (V_p_), and Prostate Imaging Reporting and Data System Version 2 (PI-RADS v2) score were determined. The apparent diffusion coefficient (ADC) was calculated from DWI. The diagnostic performance of these parameters was assessed by receiver operating characteristic (ROC) curve analysis, and those showing a significant difference between the PCa and BPH groups were combined into a multivariate logistic regression model for PCa diagnosis. Spearman’s correlation was used to analyze the relationship between Gleason score and imaging parameters.

**Results:**

The study enrolled 65 patients including 32 with PCa and 33 with BPH. Ktrans (P=0.006), Kep (P=0.001), and Vp (P=0.009) from DCE-MRI and ADC (P<0.001) from DWI could distinguish between the 2 groups when PSA was ≥10 ng/ml. PI-RADS score (area under the ROC curve [AUC]=0.705), Ktrans (AUC=0.700), Kep (AUC=0.737), Vp (AUC=0.688), and ADC (AUC=0.999) showed high diagnostic performance for discriminating PCa from BPH. A combined model based on PI-RADS score, Ktrans, Kep, Vp, and ADC had a higher AUC (1.000), with a sensitivity of 0.998 and specificity of 0.999. Imaging markers showed no significant correlation with Gleason score in PCa.

**Conclusion:**

DCE-MRI and DWI parameters can distinguish between benign and malignant prostate tumors in patients with serum PSA ≥10 ng/ml.

## Introduction

Prostate cancer (PCa) has a high morbidity and mortality (1). Prostate tumors are small, slow-growing lesions that are potentially curable at an early stage. Prostate tumor cells can undergo malignant transformation and overproliferate within a short period of time, which is associated with a poor outcome (2). Current diagnostic methods for PCa include measurement of prostate-specific antigen (PSA), fine needle aspiration biopsy, and postoperative pathologic examination. PSA is a highly expressed marker in the prostate; however, abnormally high PSA concentrations are not necessarily indicative of PCa, as serum PSA is also elevated in benign prostatic hyperplasia (BPH) (3). Thus, measurement of serum PSA lacks specificity and sensitivity for diagnosing PCa. The gold standard is biopsy but pathologic information can only be obtained after surgery or through invasive method by needle biopsy, which is unacceptable for high-risk patients. Transrectal ultrasound (TRUS) scanning is efficient for screening but is associated with complications.

Solid tumors exist in a complex microenvironment that contributes to tumor heterogeneity (4). Increased angiogenesis is correlated with tumor cell proliferation and metastasis. Dynamic contrast enhancement magnetic resonance imaging (DCE-MRI) is widely used to monitor changes in vascular permeability (5–7). Five quantitative parameters that can be extracted from DCE-MRI are the transfer constant (K_trans_), rate constant (K_ep_), fractional volume of the extravascular extracellular space (V_e_), and plasma volume (V_p_) (8–10). K_trans_ represents the diffusion rate of the gadolinium (Gd) contrast agent; Ve is the volume of Gd contrast relative to the total extravascular extracellular space volume; K_ep_ is K_trans_/V_e_; and V_p_ is calculated from the volume of Gd contrast agent in plasma. These parameters can be used to measure vessel density and the permeability of the vessel endothelium. Diffusion-weighted imaging (DWI) reflects the Brownian motion of H_2_O; the diffusion rate is used to calculate the apparent diffusion coefficient (ADC), which is directly proportional to the metabolic rate—and accordingly, the aggressivity—of the tumor. ADC has been applied to the classification of a variety of tumors including breast tumors, glioma, etc. (11) Prostate Imaging Reporting and Data System Version 2 (PI-PRADS v2) is recommended as a noninvasive method for diagnosing PCa, although it has low specificity (12).

Given the limitations of PSA, TRUS, and PI-PRADS v2, the present study investigated the clinical utility of DCE-MRI and DWI parameters for differentiating between PCa and BPH. We also established a multivariate logistic regression model that can be used to predict the malignancy of PCa.

## Materials and methods

### Patients

**Figure 1:**
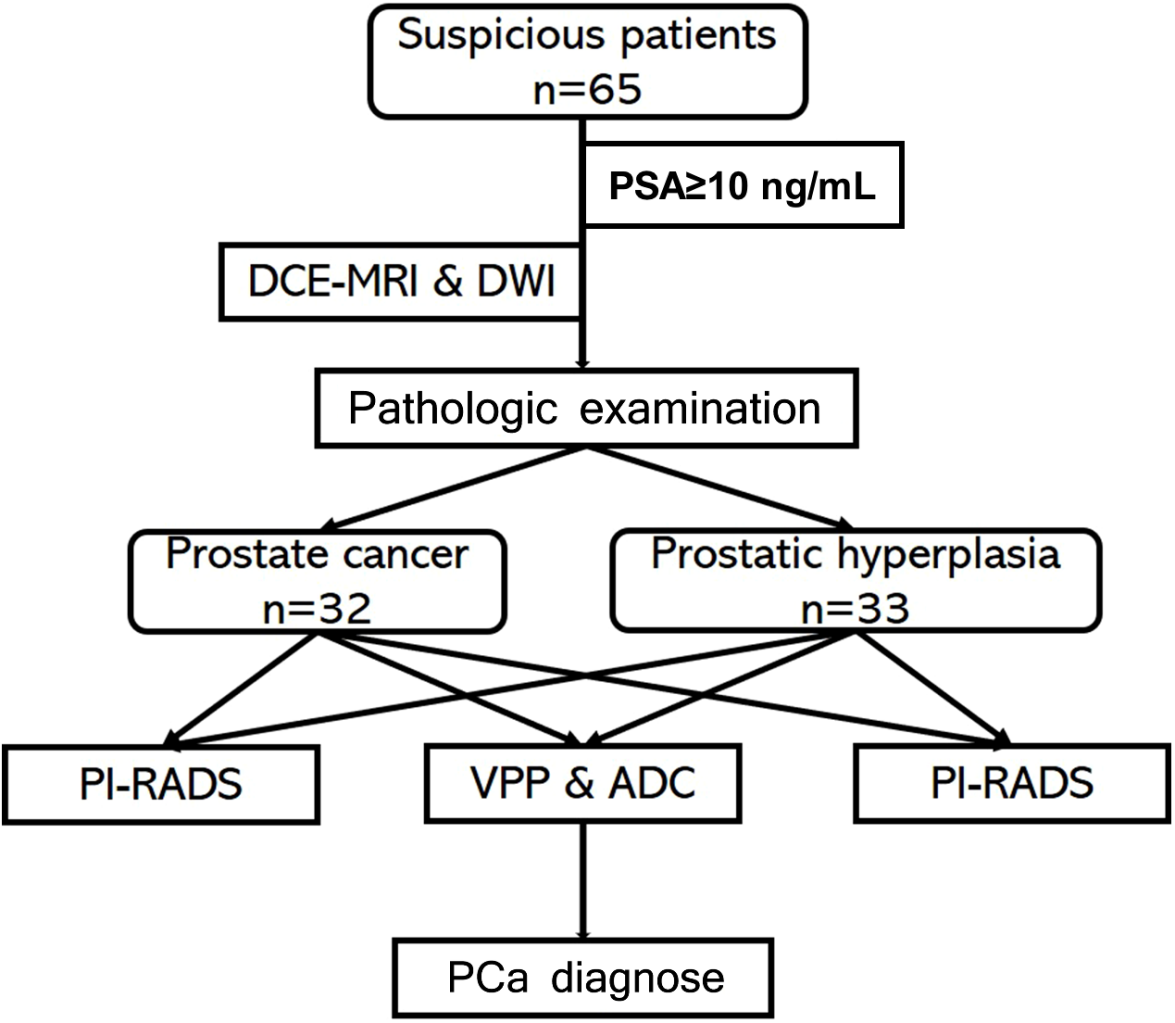
Flow diagram of the study protocol.

For this retrospective study, patients with elevated PSA and clinically suspected PCa or BPH were recruited at Henan Province Hospital of Traditional Chinese Medicine (TCM) (Zhengzhou, China) between December 2016 and October 2020. The inclusion criteria were as follows: 1) PCa or BPH confirmed by pathologic examination following ultrasound-guided puncture or surgical tumor biopsy; 2) no treatment prior to MRI scanning; 3) no MRI within 3 weeks of pathologic examination; 4) no MRI contraindications such as claustrophobia; 5) PSA ≥10 ng/ml before MRI; and 6) good image quality sufficient for diagnosis. The study was approved by the institutional review board of Henan Province Hospital of TCM. A flow diagram of the study protocol is shown in **Figure 1**. MRI scanning

All patients underwent DCE-MRI examination with a 3.0T MRI scanner (Signa HDxt; GE Healthcare, South Burlington, VT, USA). MRI scanning data no more than 1 month before surgery. The MRI protocol was set according to PI-RADS v2 (12). Patients lay in the supine position and were scanned feet-first. The 8-channel body phased surface coil (GE Healthcare) was located above the pelvis. The general MRI scan included T1-weighted imaging (T1WI), T2WI, liver acquisition with volume acceleration (LAVA), and DWI. The LAVA sequence parameters were as follows: repetition time (TR)=3.368 ms, echo time (TE)=1.672 ms, flip angle=15°, number of excitations (NEX)=1, acquisition matrix=256×192, bandwidth (BW)=244.141 Hz, field of view (FOV)=512×512, slice thickness=5, time resolution=10 s, period images=21, and scanning time=3.5 min. Before LAVA, patients were scanned with multiple fractional anisotropy (FA) sequences (FA=3°, 9°, and 12°) with only 1 phase of LAVA. Gadopentetate dimeglumine (Omniscan^®^; GE Healthcare) was injected with a high-pressure injector at a rate of 2 ml/s at 0.1 ml/kg body weight, followed by flushing with 20 ml saline solution. DWI was performed with the following parameters: b value=100 and 800, TR=5200 ms, TE=75.9 ms, FA=90, slice thickness=4, FOV=256×256, NEX=6, acquisition matrix=96×130, and BW=1953.12 Hz.

### Imaging data analysis

An ADC map was obtained from the DWI scan. Two radiologists with 5–10 years of experience delineated the suspected lesions in all slices on ADC maps by comparing T1WI and T2WI data. At same time, seminal vesicles, vessels, calcification, hemorrhage, and artifacts were excluded from the region of interest (ROI). Each ROI was segmented twice to calculate the mean ADC value. DCE-MRI images were input into Omni-Kinetics v2.1.0.R software (GE Healthcare, Shanghai, China) (9). The T1 map was generated, and T10 was calculated from the multi-FA sequence (10). We selected the femoral artery to calculate arterial input function (AIF) of normal vessels and obtained a concentration–time curve (10). We used the Tofts model (13) to calculate the vascular permeability parameters K_trans_, K_ep_, V_e_, and V_p_ and generated a map. In order to obtain the vascular permeability parameter values in lesions, the ROI was marked as the lesion in all DCE-MRI images to ensure that the ROI could be identified in the biopsy specimens (**Figure 1**).

**Figure 2:**
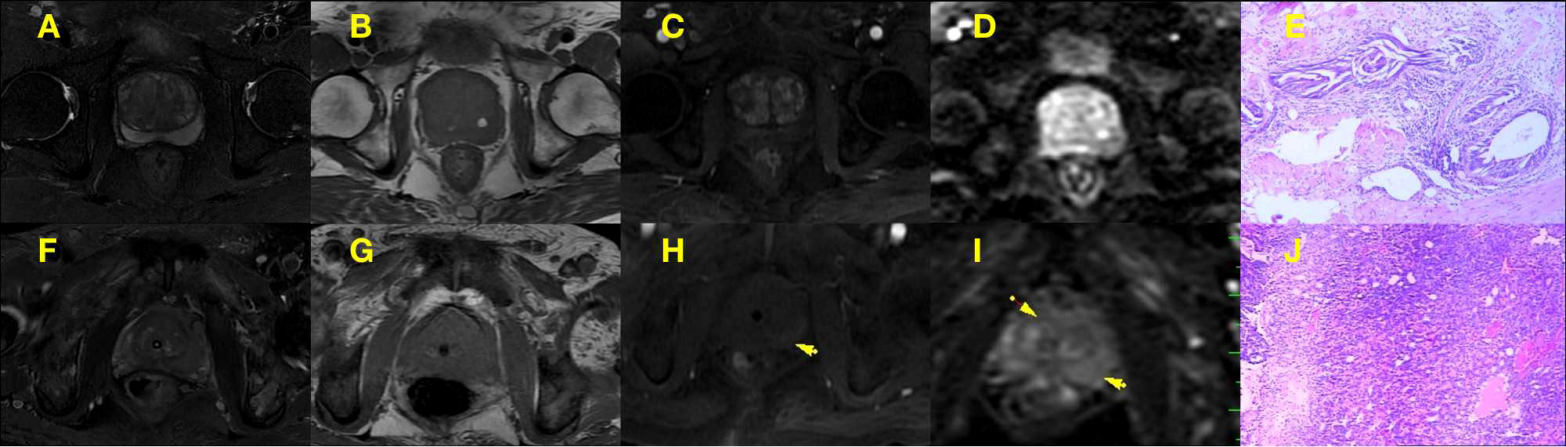
**(A–E)** PI-RADS 2 scored alterations. An example of PI-RADS 2 lesion confirmed at biopsy as pathologic examination of BPH patient **(E)**, multi-parameter MRI showed hypoint T2 signal intensity in the peripheral zone, iso and hyper T2 signal intensity in the central gland**(A)**, iso and hyper T1 signal intensity in the central gland **(B)**, normal diffusivity at b800 DWI **(C)** with contrast enhancement **(D)**, that the peripheral zone in the multi parameter MRI showed 2 points and the central gland in the multi parameter MRI showed 2 points, DWI showed 1 points. **(F–J)** PI-RADS 5 scored alterations. An example of PI-RADS 5 lesion confirmed at biopsy as pathologic examination of PCa patient **(J)**, multi-parameter MRI showed so and hyper T2 signal intensity in the central gland and peripheral zone **(F)**, iso and hyper T1 signal intensity in the central gland **(G)**, hyperintensity signal at b800 DWI **(H)** with contrast enhancement **(I)**, that the T2WI showed 5 points and DWI showed 5 points.

Two relatively experienced radiologists (HS and FD) who were blinded to the clinical information of patients retrospectively and independently evaluated the images and assigned a PI-RADS score to suspicious lesions according to PI-RADS v2 guidelines (12); any disagreements were resolved by a third senior radiologist. (**Figure 2**).

### Pathologic examination

All patients with abnormal PSA underwent a pathologic examination. Tissue samples were obtained from 24 patients who underwent surgery and 41 who underwent conventional 6 + 4- or 6 + 3-needle TRUS-guided prostate biopsy (6 standard needle points, 2 points to side of the peripheral area, and 1 or 2 points to the suspicious area)^.14^ The tissue specimens were fixed in 4% paraformaldehyde for 1 week at 24°C and then embedded in paraffin. The tissue blocks were cut into sections that were stained with hematoxylin and eosin and examined under a light microscope by a pathologist with 5 years of experience. A Gleason score—which was calculated as the sum of primary and secondary patterns and ranged from 2 to 10, with a higher score indicating poorer differentiation (14)—was assigned to each sample.

### Statistical analysis

The Mann–Whitney U test or t test was performed with Prism 8 software (GraphPad, La Jolla, CA, USA). Vascular permeability parameters and ADC are presented as mean ± standard deviation. Receiver operating characteristic (ROC) curve analysis was performed with MedCalc software (MedCalc, Ostend, Belgium). After calculating the maximum Youden index, the area under the ROC curve (AUC) was determined and a cutoff value was obtained. Spearman correlation analysis was used to assess the relationship between variables. Significant parameters were used to construct the multivariate logistic regression model. The diagnostic performance of the model was evaluated by AUC analysis. Differences with P values <0.05 were considered statistically significant.

## Results

### Clinical characteristics of patients

Five patients were excluded from the analysis because of poor DCE-MRI image quality; 7 were excluded because serum PSA was unavailable; and 25 were excluded because they had not undergone a pathologic examination. Ultimately, 65 patients met the inclusion criteria, including 32 with PCa and 33 with BPH. PI-RADS scores differed significantly between the 2 groups (P=0.003; **Table 1**). The Gleason score distribution was as follows: 5 points, n=1; 6 points, n=2; 3 + 4 points, n=8; 4 + 3 points, n=9; 8 points, n=7; 9 points, n=3; and 10 points, n=2 (**Table 1** and **Figure 2**).

**Table 1:** Patients’ demographic information.

Variable	PCA n = 32	BPH n = 33	P value^a^
	n = 32	n = 33	
Age, years (mean ± SD)	64.5 ± 6.2	59.0 ± 8.4	0.013^b^
PI-RADS			0.003^b^
1	3	14	
2	0	2	
3	14	7	
4	14	10	
5	2	0	
Gleason score			
5	1		
6	2		
3+4	8		
4+3	9		
8	7		
9	3		
10	2		

^a^P value with the Mann–Whitney U test; ^b^significant difference.

BPH, benign prostatic hyperplasia; PCa, prostate cancer; PI-RADS, Prostate Imaging Reporting And Data System.

### DCE-MRI parameters and ADC in PCa and BPH patients

**Figure 3:**
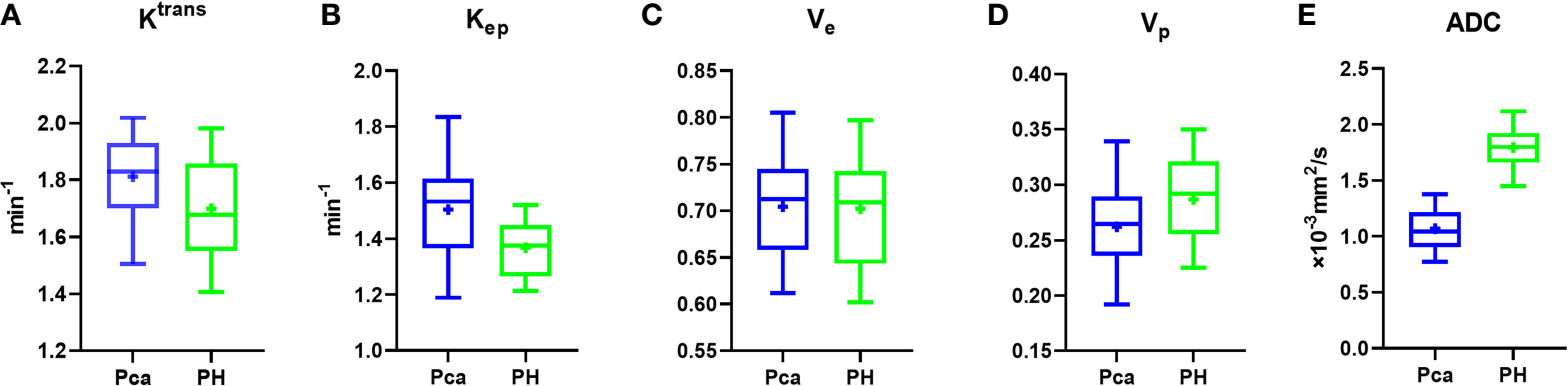
Box plot of vascular permeability parameters and ADC. **(A–E)** K_trans_ **(A)**, K_ep_ **(B)**, and ADC **(E)** differed significantly between PCa and BPH patients, whereas V_e_ **(C)** and V_p_ **(D)** were similar between the 2 groups.

DCE-MRI parameters in PCa patients were as follows: K_trans_, 1.811 ± 0.128 min^−1^; K_ep_, 1.504 ± 0.170 min^−1^; V_e_, 0.704 ± 0.052; V_p_, 0.261 ± 0.031; and ADC, (1.069 ± 0.177)×10^−3^ mm (2)/s (**Table 2**). The following 4 parameters in the BPH group showed a significant difference compared to the PCa group: K_trans_ (1.698 ± 0.169 min^−1^, P=0.006; **Figure 3A**); K_ep_ (1.367 ± 0.095 min^−1^, P=0.001; **Figure 3B**); V_p_ (0.286 ± 0.036, P=0.009; **Figure 3D**), and ADC ([1.794 ± 0.180]×10^−3^ mm(2)/s, P<0.001; **Figure 3E** and **Table 2**). V_e_ in the BPH group (0.702 ± 0.056) did not differ significantly from the value in the PCa group (P=0.911; **Table 2** and **Figure 3C**).

**Table 2:** DCE-MRI parameters and ADC for PCa and BPH groups.

Parameter	PCa	BPH	P value^a^
DCE-MRI			
K_trans_, min^−1^	1.811 ± 0.128	1.698 ± 0.169	0.006^b^
K_ep_, min^−1^	1.504 ± 0.170	1.367 ± 0.095	0.001^b^
V_e_	0.704 ± 0.052	0.702 ± 0.056	0.911
V_p_	0.261 ± 0.031	0.286 ± 0.036	0.009^b^
ADC, 10^−3^ mm^2^/s	1.069 ± 0.177	1.794 ± 0.180	<0.001^b^

Data represent mean ± SD. ^a^P value with the Mann–Whitney U test; ^b^significant difference.

ADC, apparent diffusion coefficient; BPH, benign prostatic hyperplasia; DCE-MRI, dynamic contrast-enhanced magnetic resonance imaging; K_ep_, rate constant; K_trans_, transfer constant; PCa, prostate cancer; V_e_, fractional volume of the extravascular extracellular space; V_p_, plasma volume.

### Performance of imaging parameters and PI-RADS in the differential diagnosis of PCa vs BPH

**Figure 4:**
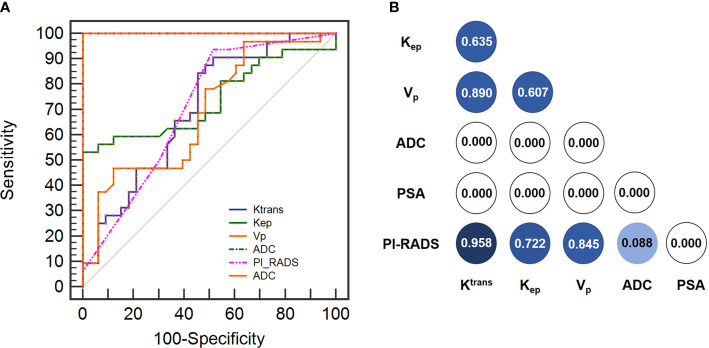
**(A)** Diagnostic performance of imaging parameters (K_trans_, K_ep_, Vp and ADC) and PI-RADS score in discriminating between PCa and BPH based on ROC curve analysis. **(B)** Delong analysis among imaging paramters (Ktrans, Kep, Vp and ADC) and PI-RADS score in discrimination between PCa and BPH.

Imaging parameters and PI-RADS score showed high diagnostic performance in discriminating between PCa and BPH patients, with AUCs of 0.705 for K_trans_, 0.700 for K_ep_, 0.737 for V_p_, 0.688 for ADC, and 0.999 for PI-RADS score (**Table 3** and **Figure 4A**). The sensitivity values of PI-RADS, K_trans_, and ADC were higher than those of K_ep_ and V_p_, whereas the specificity values of K_ep_, V_p_, and ADC were higher than those of PI-RADS and K_trans_ (**Table 3**). Given the significant differences in PI-RADS score and imaging parameters between the PCa and BPH groups (PI-RADS, P=0.0009; K_trans_, P=0.0022; K_ep_, P=0.0003; V_p_, P=0.0049; ADC, P<0.0001; **Table 3**), we compared their diagnostic performance with the Delong test but found no significant difference between PI-RADS and K^trans^ (P=0.958; **Figure 4B**), K_ep_ (P=0.722; **Figure 4B**), V_p_ (P=0.845; **Figure 4B**), and ADC (P=0.088; **Figure 4B**). ADC showed the highest diagnostic performance among imaging parameters (ADC vs K^trans^, K_ep_, and V_p_; P<0.001), whereas there was no significant difference in performance among DCE-MRI parameters (P≥0.05).Diagnostic performance of the combined model

**Table 3:** Diagnostic performance of imaging parameters and PI-RADS score in discriminating between PCa vs BPH.

	AUC	Sensitivity	Specificity	Youden indexJ statistic	95% CI	P value^a^
PI-RADS	0.705	0.937	0.485	0.422	0.579–0.812	0.0009^b^
DCE-MRI						
K_trans_	0.700	0.906	0.485	0.391	0.574–0.808	0.0022^b^
K_ep_	0.737	0.531	1.000	0.531	0.613–0.839	0.0003^b^
V_p_	0.688	0.469	0.879	0.347	0.560–0.797	0.0049^b^
ADC	0.999	0.998	0.999	1.000	0.945–1.000	<0.0001^b^

^a^P value with the Mann–Whitney U test; ^b^significant difference.

ADC, apparent diffusion coefficient; AUC, area under the curve; BPH, benign prostatic hyperplasia; CI, confidence interval; DCE-MRI, dynamic contrast-enhanced magnetic resonance imaging; K_ep_, rate constant; K_trans_, transfer constant; PCa, prostate cancer; PI-RADS, Prostate Imaging Reporting And Data System; V_p_, plasma volume.

**Figure 5:**
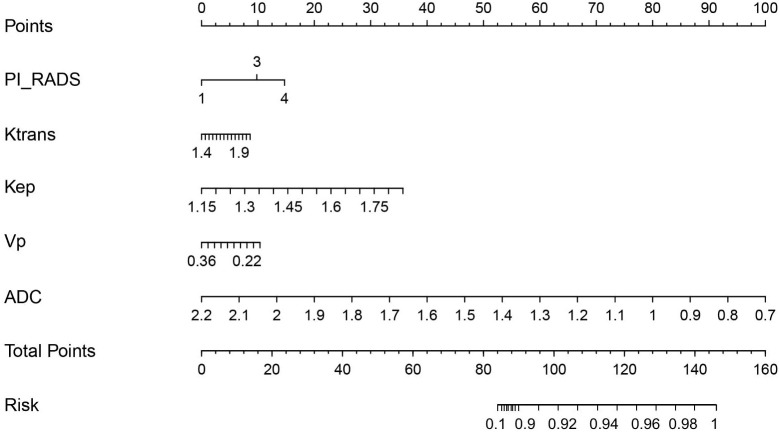
Nomogram for differentiating PCa from BPH. The nomogram was developed with PI-RADS, K_trans_, K_ep_, V_p_, and ADC. This nomogram can be used to classification of Pca from BPH. Before biopsy or surgery performed, patients underwent DCE-MRI and DWI scanning that, K_trans_, K_ep_, V_p_, ADC and PI-RADS were determined. All of these results will put into the Nomogram as follow: Points_PI_RADS will calculate by the score of Points which is vertically projected on the line of Points by PI-RADS. K_trans_, K_ep_, V_p_ and ADC should be vertically projected on the line of Points to get Points_K_trans_, Points_K_ep_, Points_V_p_ and Points_ADC. After this, the Total points is the sum of Points_PI_RADS, Points_K_trans_, Points_K_ep_, Points_V_p_ and Points_ADC. After this, total points is vertically projected on the line of Risk for patients’ probability of PCa.

PI-RADS score and imaging parameters (K_trans_, K_ep_, V_p_, and ADC) were used to construct a multivariate logistic regression model, which distinguished between PCa and BPH with an AUC of 1.000 (95% confidence interval [CI]: 0.945–1.000; **Table 4**). The logistic regression analysis identified PI-RADS score and imaging parameters (K_trans_, K_ep_, V_p_, and ADC) as independent predictors of PCa malignancy with high sensitivity (0.983) and specificity (0.999) (**Table 4**). The model incorporating the above independent predictors is presented as a nomogram (**Figure 5**).

**Table 4:** Diagnostic performance of DCE-MRI parameters, ADC, and the combined model.

	Coefficient	OR	AUC	95% CI lower	95% CI upper
Intercept	−23.465				
PI-RADS	9.929	1491.79			
ADC	−134.766	9.52×10^−34^			
K_trans_	23.988	42909.20			
K_ep_	114.729	1.27×10^27^			
V_p_	−87.606	3.21×10^−13^			
Nomogram			1.000	0.945	1.000

ADC, apparent diffusion coefficient; AUC, area under the curve; CI, confidence interval; DCE-MRI, dynamic contrast-enhanced magnetic resonance imaging; K_ep_, rate constant; K_trans_, transfer constant; OR, odds ratio; PI-RADS, Prostate Imaging Reporting And Data System; V_p_, plasma volume.

### Correlation between Gleason stage and DCE-MRI parameters and ADC

A Spearman correlation analysis was performed to examine the relationship between imaging parameters and Gleason stage. The values of DCE-MRI parameters of the tumor region were negatively correlated with Gleason stage, but not significantly (K_trans_, r=−0.220, P=0.226; K_ep,_ −0.177, P=0.332; V_e_, −0.080, P=0.663; and V_p,_ −0.058, P=0.754; **Table 5**). The ADC of the tumor region was positively correlated with Gleason stage (r=0.145), but this association was also nonsignificant (P=0.430; **Table 5**).

**Table 5:** Spearman correlations between imaging parameters and Gleason stage.

	K_trans_	K_ep_	V_e_	V_p_	ADC
r	−0.220	−0.177	−0.080	−0.058	0.145
P value	0.226	0.332	0.663	0.754	0.430

ADC, apparent diffusion coefficient; K_ep_, back flow rate constant; K_trans_, volume transfer rate constant; V_e_, extravascular extracellular space volume fraction; V_p_, plasma volume fraction.

## Discussion

The results of this study demonstrate that DCE-MRI and DWI parameters can differentiate between benign and malignant prostate tumors when serum PSA is ≥10 ng/ml. Thus, K_trans_ (AUC=0.700), K_ep_ (AUC=0.737), V_p_ (AUC=0.688), and ADC (AUC=0.999) can be used as imaging biomarkers to evaluate PCa along with PI-RADS score. In order to improve diagnostic specificity and sensitivity, we established a multivariate logistic regression model to predict tumor malignancy based on 3 vascular permeability parameters; ADC and PI-RADS differed significantly between PCa and BPH patients. We also established a nomogram to visualize the multivariate logistic regression model, which showed higher AUC (1.000) and higher sensitivity (0.983) and specificity (0.999). However, vascular permeability parameters and ADC did not show significant correlations with Gleason stage in PCa patients.

PCa is diagnosed as high risk when PSA is ≥10 ng/ml (15). The goal of PSA screening is to classify PCa at an earlier stage. However, a diagnosis cannot be made based solely on PSA level. The typical PSA level in PCa is 4 ng/ml; this is too low for biopsy, which is usually recommended for PSA levels of 4–10 ng/ml (16). The AUC of PSA (4–10 ng/ml) for differentiating between PCa and BPH was 0.708, with a sensitivity of 0.837 and specificity of 0.583 (17); thus, screening based on PSA can lead to overdiagnosis and overtreatment (18). Some DCE-MRI studies have shown that the vascular permeability parameters K_trans_ and K_ep_ were higher in PCa than in BPH (19, 20), which is supported by our results. However, the latter study also showed that V_e_ and V_p_ differed between these 2 groups, which was confirmed by another report (20) and is contrary to our findings. Ours is the first investigation of the diagnostic performance of DCE-MRI and DWI parameters in PCa when serum PSA is ≥10 ng/ml, which is more closely associated with PCa risk than any specific marker (16). Vascular permeability values reported in PCa vary across the literature, even considering a 95% CI; this may be attributable to the reference vessel for AIF (21), parameters of the DCE-MRI sequence (eg, temporal resolution) of different instrument manufacturers, and interindividual differences among patients (22). There is accumulating clinical evidence that DWI is a useful tool for the quantitative assessment of tumor characteristics and prognosis (23, 24). ADC reflects the Brownian motion of H_2_O, which is constantly interacting with other molecules in the tissue microenvironment. Proliferating tumor cells can inhibit H_2_O movement, resulting in a change in the ADC value; therefore, ADC is used as a marker for tumor malignancy and prognosis (25, 26) and to discriminate between cancer and noncancer tissue (27). In the current study, ADC values differed significantly between PCa and BPH, suggesting that aggressive tumors block the diffusion of H_2_O to a greater extent than those that are benign. DWI of 60 patients revealed that ADC was an independent factor that could distinguish between BPH and transition zone cancer (1.32 ± 0.19 vs 0.89 ± 0.17 mm(2)/s; P<0.001) (19). Compared to vascular permeability parameters, ADC showed better diagnostic performance in detecting PCa, with an AUC of 0.999, sensitivity of 0.998, and specificity of 0.999.

PI-RADS v2 is a noninvasive method to predict PCa with low specificity (28), which was confirmed in our study. To improve sensitivity and specificity, we constructed an imaging-based model combining PI-RADS score, ADC, K_trans_, K_ep_, and V_p_ that was visualized as a nomogram and had a sensitivity of 0.983 and specificity of 0.999; these were higher than the corresponding values for PI-RADS score and vascular permeability parameters. In fact, ADC also showed high sensitivity (0.998) and specificity (0.999) in our study. Multiparameter (mp)MRI is increasingly recommended for noninvasive PCa screening (17). A combined model based on mpMRI showed higher diagnostic performance compared to a single imaging parameter (17). Additional studies with a larger sample size are needed to determine whether ADC can serve as an imaging biomarker for the differentiation of PCa from BPH.

In this study, we have built a multiple logistic model to classification of Pca and BPH with an amazing diagnostic discrimination (AUC=1.000) by combining PI-RADS score, ADC, K_trans_, K_ep_, and V_p_. To provide the clinician with a simplified quantitative tool to predict individual probability of PCa, Nomogram was drawn on the basis of imaging-based model combining PI-RADS score, ADC, K_trans_, K_ep_, and V_p_. For example, there were a representative case to illustrate the discriminative ability of nomograms for the classification of Pca. A 62-year-old man with PSA=12 ng/ml. After MRI scanning, his PI-RADS score was 3, K_trans_ was 1.723 min^-1^, K_ep_ was 1.43 min-1, V_p_ was 0.367, ADC was 1.025x10^−3^ mm(2)/s. After put these value into the nomogram, we have got the score of PI_RADS (Points=9.5), K_trans_ (Points=7.3), K_ep_(Points=30.1), V_p_ (Points=0.1)and ADC (Points=79.1) that the total points is 126. After vertically projected on the line of Risk the probability of PCa is 96%.

We also analyzed the correlation between imaging parameters and Gleason score, which is used for histologic staging of PCa and is an important prognostic factor. A significant negative correlation between ADC and Gleason score was reported; this may be explained by the fact that the high proliferation rate of tumor cells leads to a higher cell density, which reduces extracellular space and restricts H_2_O movement (19). However, we did not observe a significant correlation between ADC and Gleason score, which may be due to the small number of patients in the PCa group.

The present study had some limitations. Firstly, as the patients were from a single hospital it is unclear whether our findings are generalizable to all PCa patients. Secondly, ROI segmentation was performed by 2 experienced radiologists, but we did not evaluate intraobserver differences. Multicenter studies addressing these shortcomings are needed to achieve a higher level of evidence.

## Conclusion

K_trans_ and K_ep_ in DCE-MRI and ADC in DWI can be used as imaging biomarkers to distinguish PCa from BPH. A multivariate logistic regression model combining these 3 parameters showed good diagnostic performance for PCa. Thus, DCE-MRI and DWI are useful noninvasive diagnostic tools that can guide management strategies for PCa patients.

## Data availability statement

The raw data supporting the conclusions of this article will be made available by the authors, without undue reservation.

## Ethics statement

The studies involving human participants were reviewed and approved by Henan Province Hospital of TCM. The patients/participants provided their written informed consent to participate in this study.

## Author contributions

HS, FD, and TW designed the study. HS, FD, and YL performed the experiments. QL, XL, and HS analyzed the data. HS, FD, and TW wrote the manuscript. TW revised the manuscript and supervised the study. All authors contributed to the article and approved the submitted version.
