# Supplementary figures and images for: DCE-MRI and DWI can differentiate benign from malignant prostate tumors when serum PSA is ≥10 ng/ml
Source: Front Oncol. 2022 Dec 12;12:925186. doi: 10.3389/fonc.2022.925186 (PMC9792168; doi:10.3389/fonc.2022.925186)

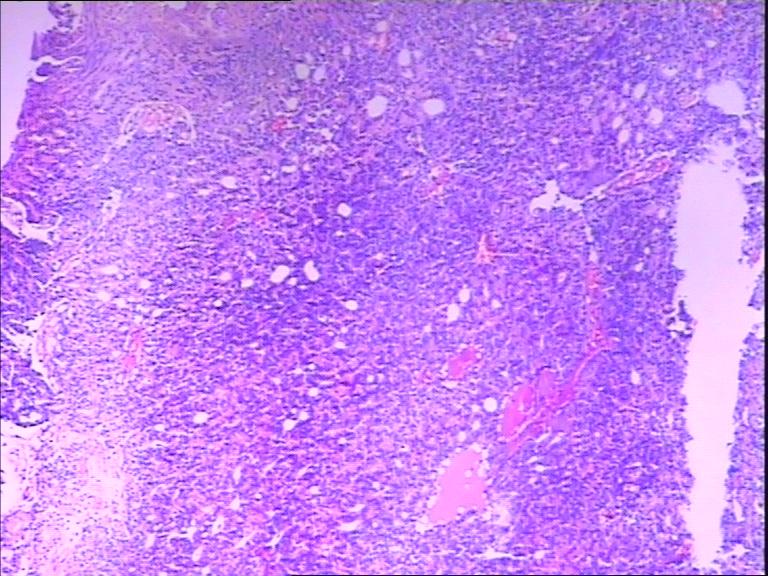

Supplement: Supplementary file 1 [file DataSheet_1.zip › down/τùàτÉå.jpg]

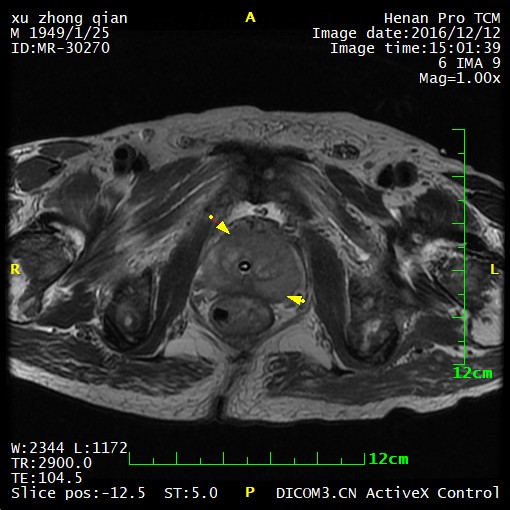

Supplement: Supplementary file 1 [file DataSheet_1.zip › down/MRI/σ╛«Σ┐íσ¢╛τëç_20210114110958.jpg]

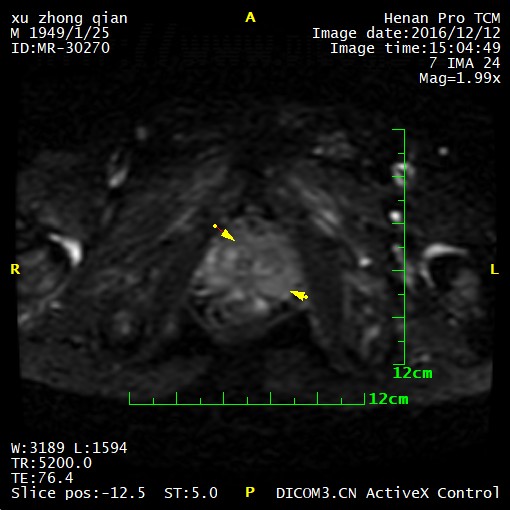

Supplement: Supplementary file 1 [file DataSheet_1.zip › down/MRI/σ╛«Σ┐íσ¢╛τëç_20210114111014.jpg]

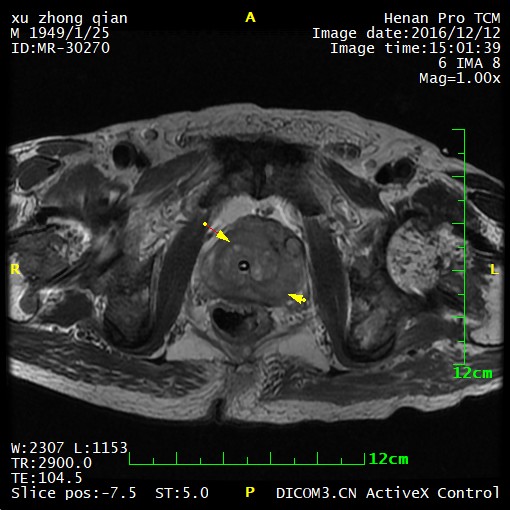

Supplement: Supplementary file 1 [file DataSheet_1.zip › down/MRI/σ╛«Σ┐íσ¢╛τëç_20210114110906.jpg]

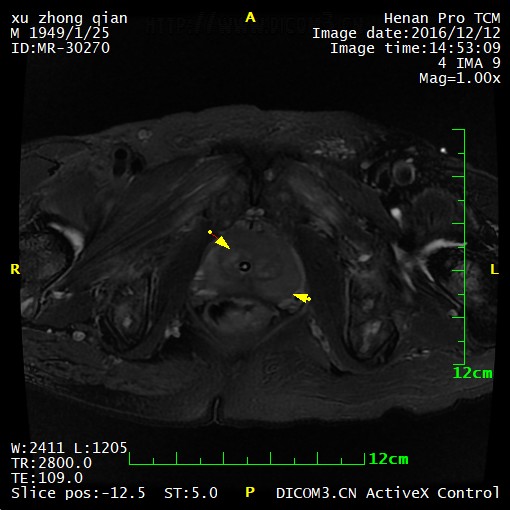

Supplement: Supplementary file 1 [file DataSheet_1.zip › down/MRI/σ╛«Σ┐íσ¢╛τëç_20210114111006.jpg]

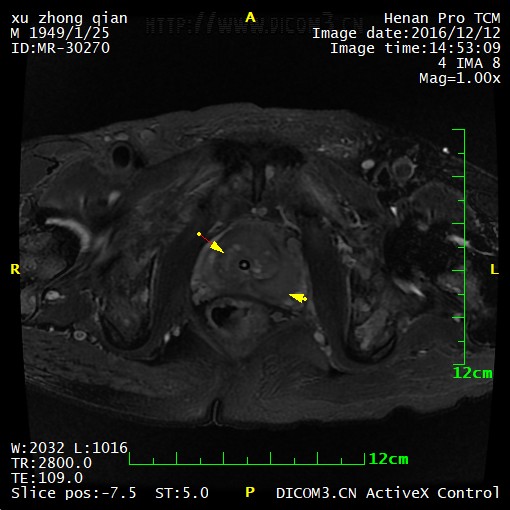

Supplement: Supplementary file 1 [file DataSheet_1.zip › down/MRI/σ╛«Σ┐íσ¢╛τëç_20210114110937.jpg]

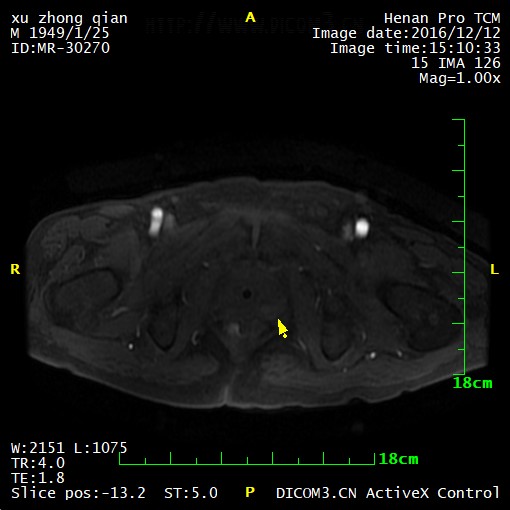

Supplement: Supplementary file 1 [file DataSheet_1.zip › down/MRI/σ╛«Σ┐íσ¢╛τëç_20210114111019.jpg]

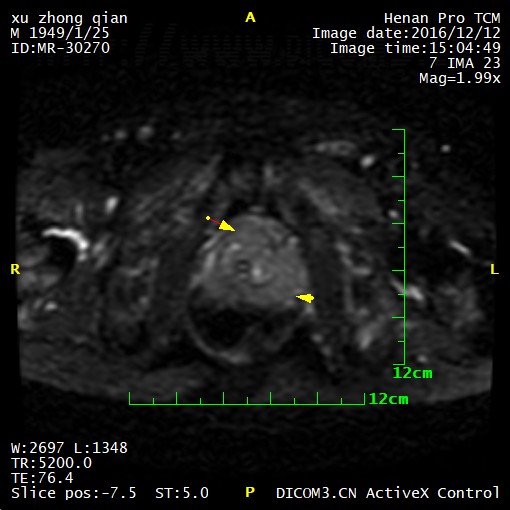

Supplement: Supplementary file 1 [file DataSheet_1.zip › down/MRI/σ╛«Σ┐íσ¢╛τëç_20210114110941.jpg]

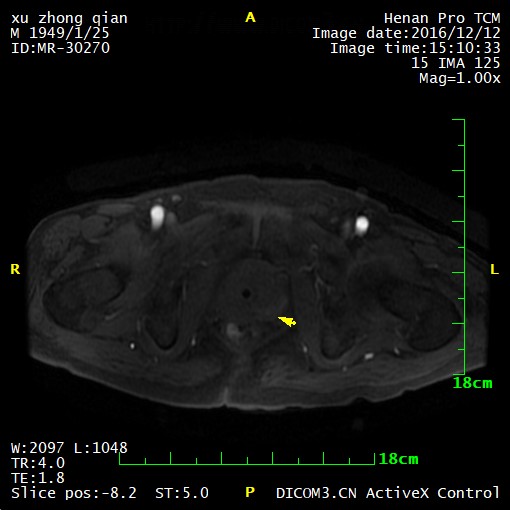

Supplement: Supplementary file 1 [file DataSheet_1.zip › down/MRI/σ╛«Σ┐íσ¢╛τëç_20210114110953.jpg]
